# Multiple benefits of parecoxib sodium in perioperative applications

**DOI:** 10.1097/JS9.0000000000003197

**Published:** 2025-08-21

**Authors:** Shou-Meng Geng, Hai-Xia Wang, Hai-Cang Xu

**Affiliations:** a Department of Emergency Internal Medicine, The Affiliated Hospital of Qingdao University, Qingdao, Shandong, China; b Department of Pathology Department, The Affiliated Hospital of Qingdao University, Qingdao, Shandong, China

*Dear Editor,*

We appreciate the authors’ work in the recent article “Effect of parecoxib on postoperative delirium inpatients with hyperlipidemia: a randomized, double-blind, single-center, superiority trial”^[1]^. This study is a randomized, double-blind, single-center, superiority trial aimed at evaluating the preventive effect of parecoxib on postoperative delirium (POD) in patients with hyperlipidemia. The results showed that the incidence of POD in the parecoxib group was significantly reduced (13.72% vs. 26.11%, HR 0.491, *P* < 0.001), and PTGS2 levels, neutrophil counts, white blood cell counts, and pain scores were all significantly improved. Mediation analysis indicated that parecoxib indirectly reduces the risk of POD by inhibiting PTGS2 and alleviating inflammatory responses. The study suggests that the anti-inflammatory and analgesic effects of parecoxib may be the key mechanisms for its prevention of POD. Building upon this foundation, we offer the following insights for discussion

## Insight 1: The long-term cognitive protective effect of parecoxib has not been fully explored

This study focuses on the incidence of POD within 3 days after surgery but does not evaluate the impact of parecoxib on long-term cognitive function. Existing evidence shows that POD is associated with an increased risk of postoperative cognitive dysfunction and dementia, and the neuroprotective mechanisms of parecoxib (such as inhibiting neuroinflammation) may be beneficial for long-term cognition^[2]^. For example, animal experiments have shown that COX-2 inhibitors can reduce β-amyloid deposition, but this study did not track patients’ cognitive changes after 3 months (only assessed by TICS-M telephone interview). Future studies should extend the follow-up period and combine brain imaging or cerebrospinal fluid biomarkers (such as tau protein) to verify whether parecoxib has the potential to delay neurodegeneration. In addition, patients with hyperlipidemia often have comorbid cerebrovascular diseases, and whether parecoxib can further protect cognition by improving cerebral blood perfusion still needs to be verified by multi-center studies.

## Insight 2: Dynamic monitoring of inflammatory mediators and individualized intervention strategies

The study only detected PTGS2 and inflammatory cell levels before surgery and on the first day after surgery, failing to reflect the dynamic changes of perioperative inflammation. The occurrence of POD is related to the timing of inflammatory responses. For example, the peak of IL-6 within 24 hours after surgery is significantly associated with the risk of delirium. Increasing multiple samplings during surgery and 48 hours after surgery may more accurately identify high-risk patients. In addition, subgroup analysis showed that parecoxib was more effective in patients with higher PTGS2 levels (*P* < 0.001), suggesting that baseline inflammation may affect the efficacy. In the future, an “inflammatory phenotype stratification” strategy can be explored: for patients with high inflammatory load (e.g., PTGS2 > 43.07 ng/mL, Figure 5A), intensive anti-inflammatory treatment (such as combining IL-1 receptor antagonists) can be applied, while intervention can be reduced for patients with low load. This individualized scheme may optimize resource allocation and reduce the risk of drug side effects (such as gastrointestinal toxicity of NSAIDs).

## Insight 3: Economic benefits and clinical translation barriers of parecoxib

The study did not analyze the cost-effectiveness of parecoxib, but its clinical promotion needs to balance economy and efficacy. As a selective COX-2 inhibitor, parecoxib is more expensive than traditional NSAIDs, but its benefits in reducing POD (such as shortening hospital stay and reducing nursing costs) have not been quantified. For example, patients with POD have an average prolongation of hospital stay by 2.3 days^[3]^, and the incidence of POD in the parecoxib group is reduced by 12.39%, which can theoretically save medical expenses. However, the study population was patients undergoing gastrointestinal surgery (with a high complication rate), and it is unclear whether the results are applicable to low-risk surgeries (such as hernia repair). In addition, primary hospitals in China may lack PTGS2 detection conditions (relying on ELISA, methodology section), which limits the popularization of the scheme. It is suggested that subsequent studies should include health economic evaluation and develop simple inflammatory markers (such as C-reactive protein) as alternative indicators to promote clinical translation. Multiple studies have shown that parecoxib sodium (a selective COX-2 inhibitor) exhibits comprehensive benefits in perioperative applications, including analgesia, anti-inflammation, and reducing the risk of POD.

In general, parecoxib sodium (a selective COX-2 inhibitor) demonstrates comprehensive benefits in perioperative applications, including analgesia, anti-inflammation, and reducing the risk of POD. By inhibiting inflammatory factors (such as TNF-α and IL-6) and upregulating anti-inflammatory mediators (such as HO-1 and IL-10), parecoxib sodium significantly reduces the incidence of POD (6.2% vs. 11–27.5%), while also decreasing opioid consumption and pain scores. Its mechanisms involve the inhibition of neuroinflammation and oxidative stress, such as reducing the levels of nerve injury markers (S-100β, NfL). In addition, preoperative administration is more effective than intraoperative or postoperative administration, with good safety and a lower adverse reaction rate compared to traditional analgesic regimens. These findings support parecoxib sodium as a core drug in multimodal analgesia, particularly suitable for high-risk elderly surgical patients.

## Data Availability

Not applicable.
